# Supplementary material for: Structural and functional insights into IZUMO1 recognition by JUNO in mammalian fertilization
Source: Nat Commun. 2016 Jul 15;7:12198. doi: 10.1038/ncomms12198 (PMC4947182; doi:10.1038/ncomms12198)
Supplement: Supplementary Information — Supplementary Figures 1-5 [file ncomms12198-s1.pdf]

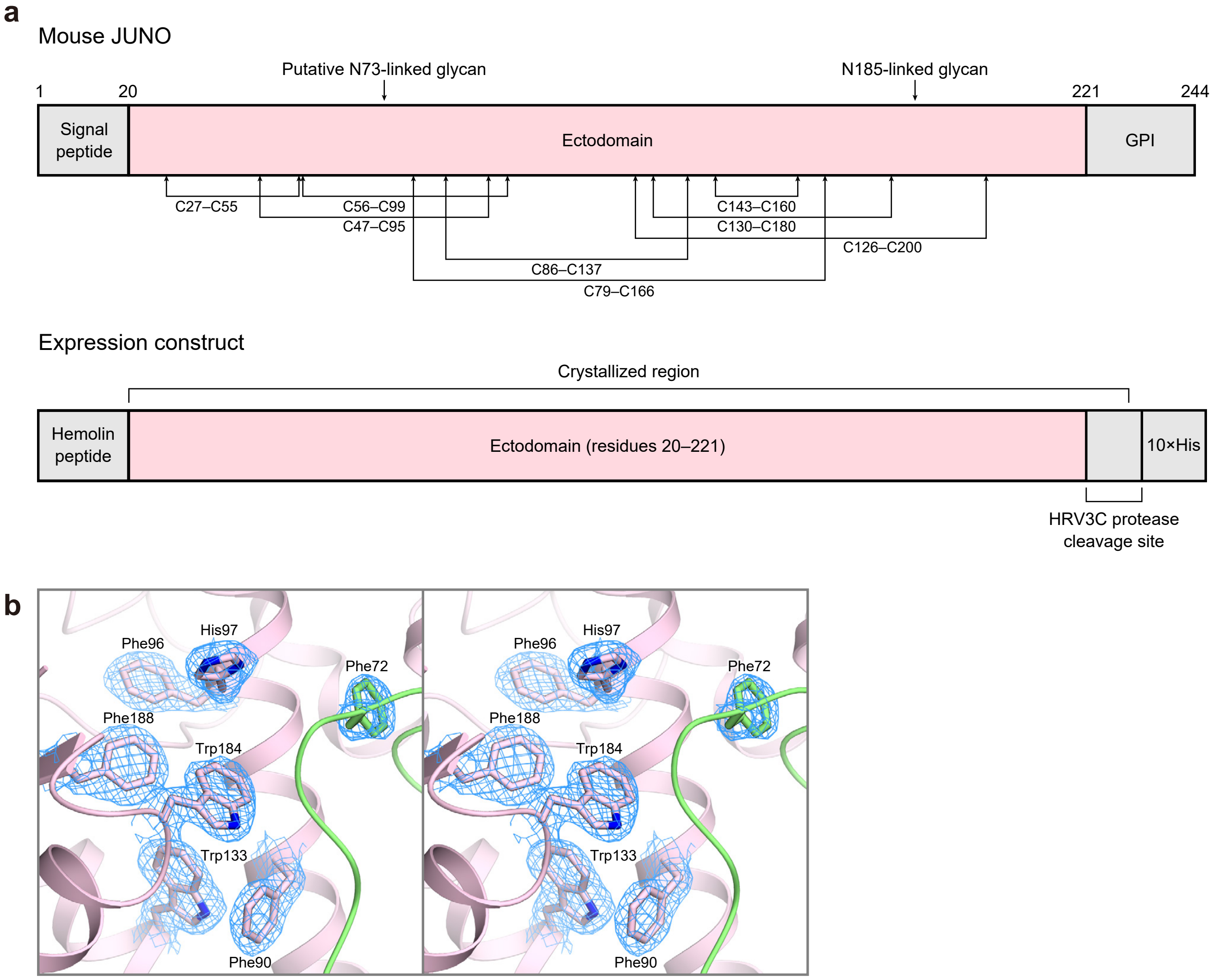

Supplementary Figure 1 | Structural determination of mouse JUNO.

(**a**) Crystallization construct. Mouse JUNO contains the N-terminal secretory signal and the C-terminal GPI-anchor attachment signal sequences. The two putative glycosylation sites (Asn73 and Asn185) and the eight conserved disulfide bonds are indicated.

(**b**) Electron density map. The $2mF_{O} - DF_{C}$ electron density map (contoured at 1.2σ) for the selected residues at the central pocket is shown as a blue mesh.

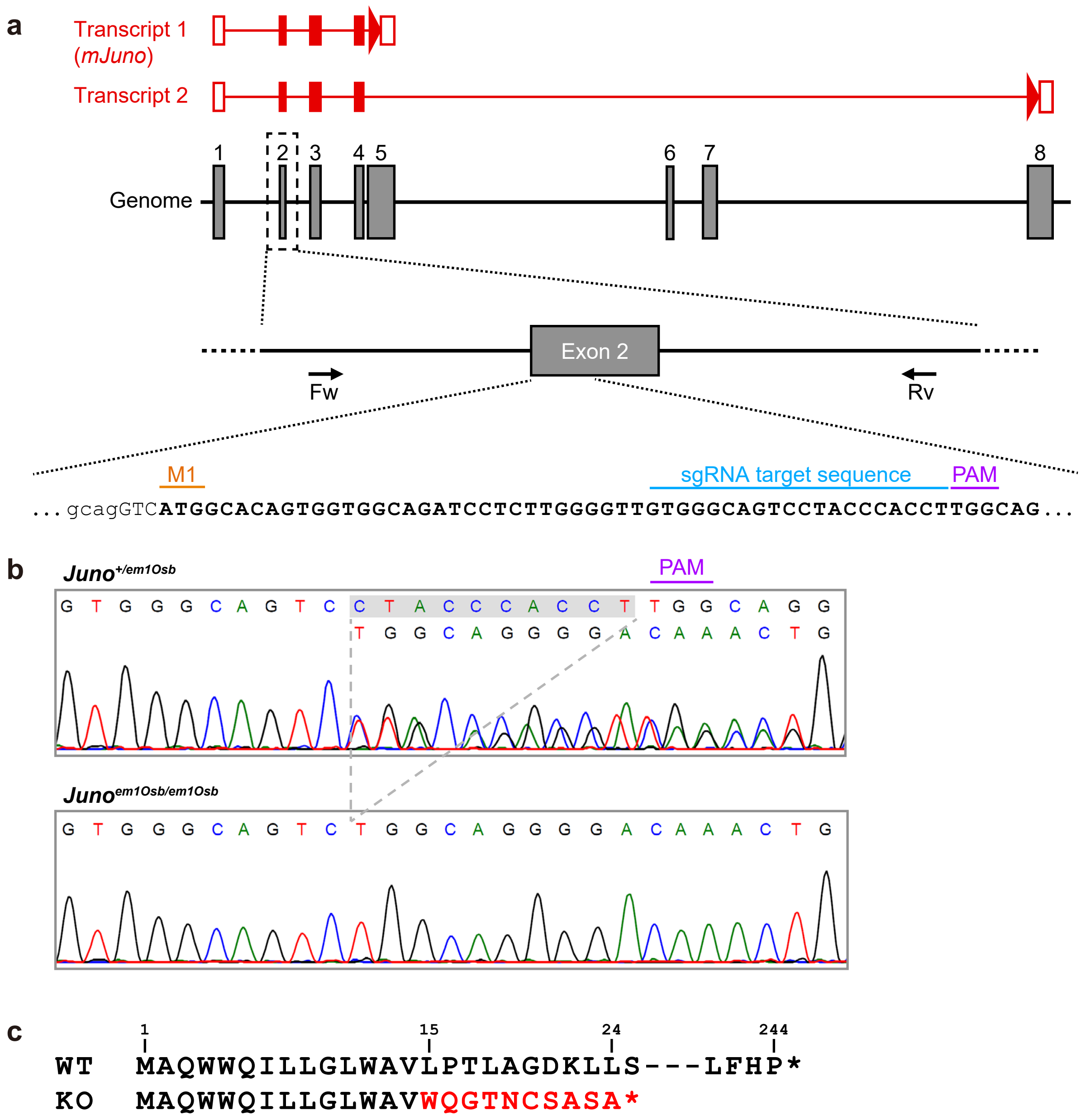

Supplementary Figure 2 | Generation of *Juno* KO mice using the CRISPR-Cas9 system.
(**a**) Exon usages of the reported protein-coding transcripts for the *Juno* gene are indicated at the top. The coding and non-coding exons are shown as solid and open bars, respectively. The sgRNA was designed to target *Juno* exon 2, encoding the common initiating methionine (orange). Forward (Fw) and reverse (Rv) primers for validation and genotyping are shown as arrows. The sgRNA guide sequence and the PAM sequence are indicated by blue and purple bars, respectively.
(**b**) Sequencing of *Juno*$^{+/em1Osb}$ (F1) and *Juno*$^{em1Osb/em1Osb}$ (F2) mice. The PAM sequence is indicated by a purple bar, and the 10-bp deleted sequence is shaded in gray.
(**c**) Amino-acid sequence of JUNO in *Juno*$^{em1Osb/em1Osb}$ mice. Frameshift mutations are colored red. Amino acid numbers are shown above the sequences. Asterisks indicate stop codons.

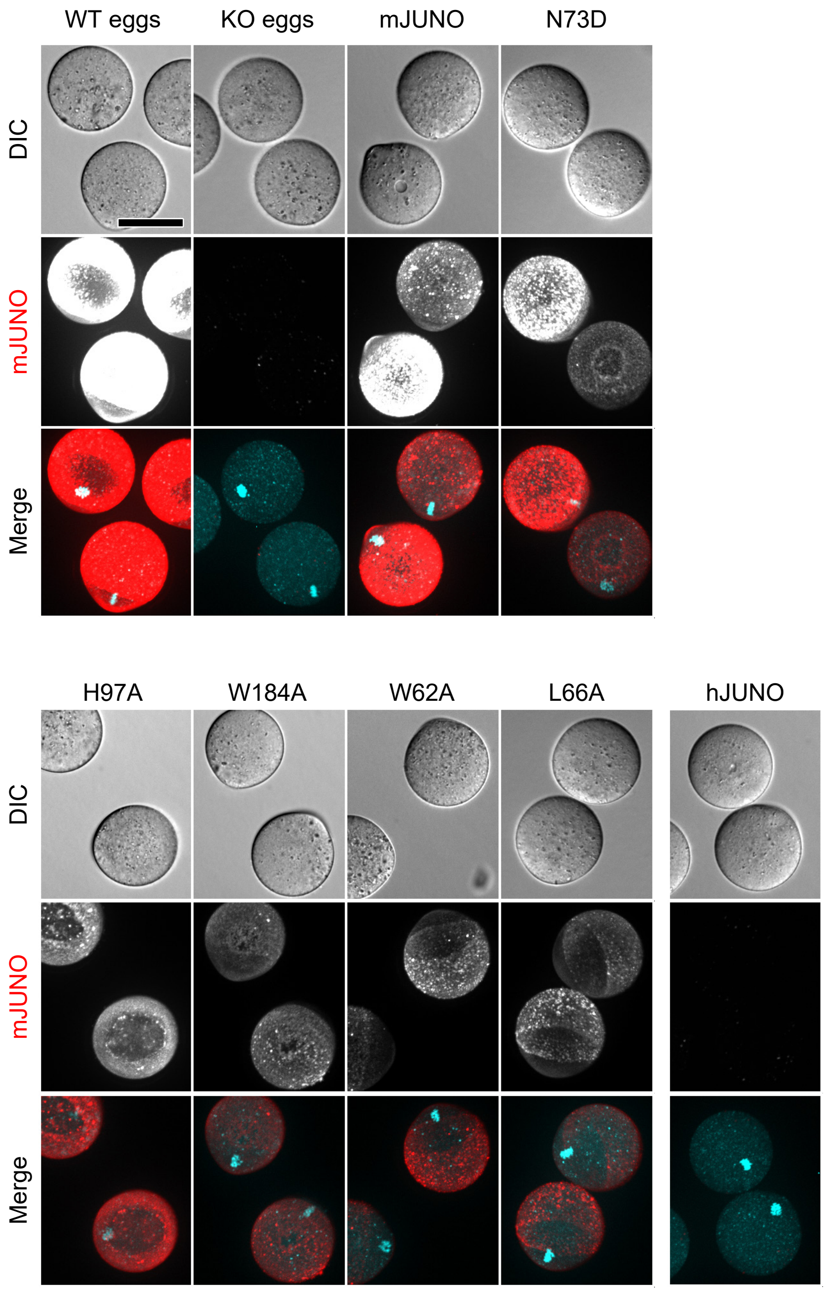

Supplementary Figure 3 | Surface expression of WT and mutant JUNOs on *Juno* KO eggs.
Immunostaining of JUNO under non-permeabilized conditions (monochrome, or red in merged images). MII chromosomal DNA stained with Hoechst 33342 is indicated in cyan in the merged images. Bar indicates 50 µm.

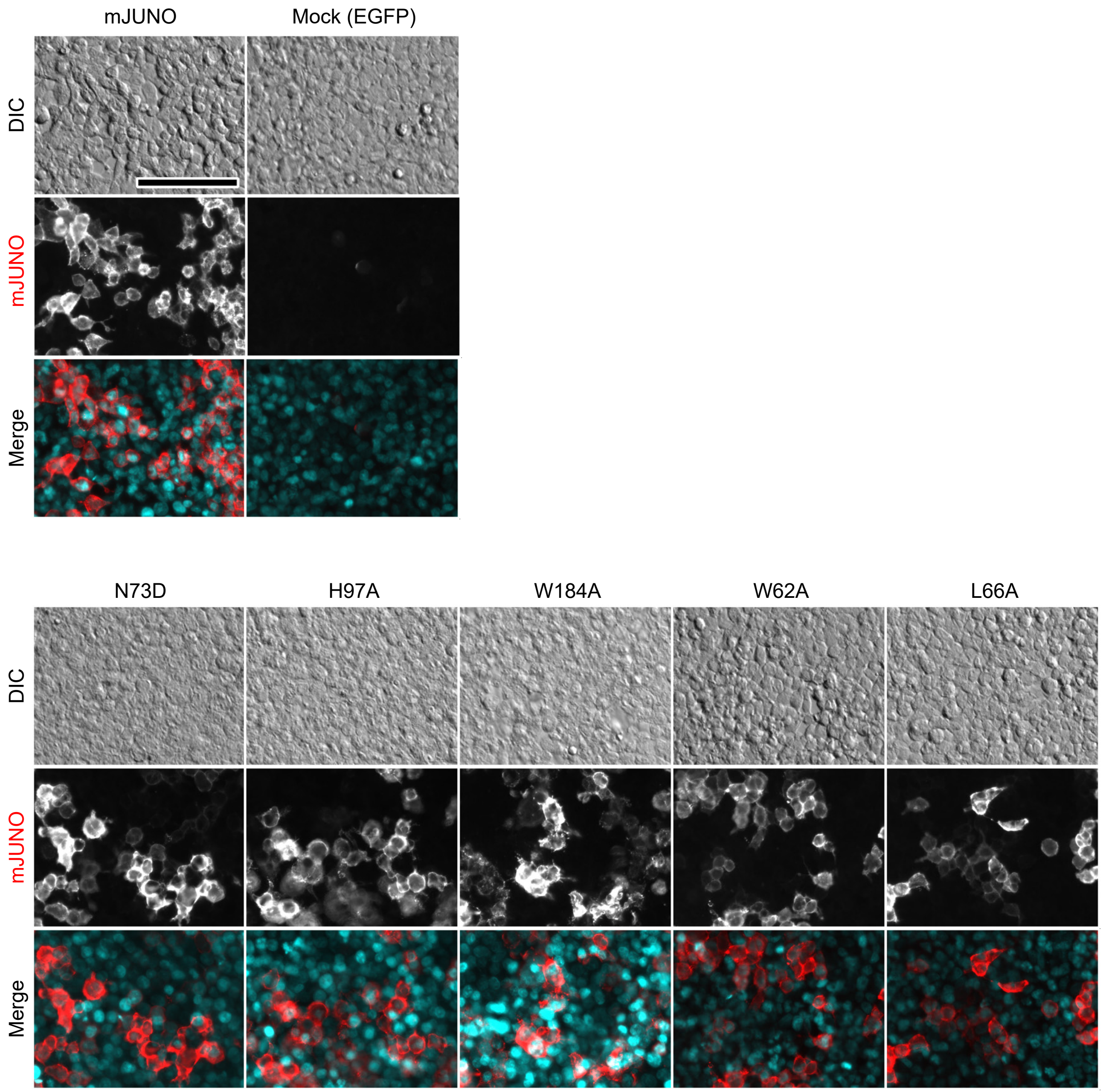

Supplementary Figure 4 | Surface expression of mJUNOs on HEK293T cells.
Immunostaining of JUNOs under non-permeabilized conditions (monochrome, or red in merged images). Nuclear DNA stained with Hoechst 33342 is indicated in cyan in merged images. Bar indicates 100 µm.

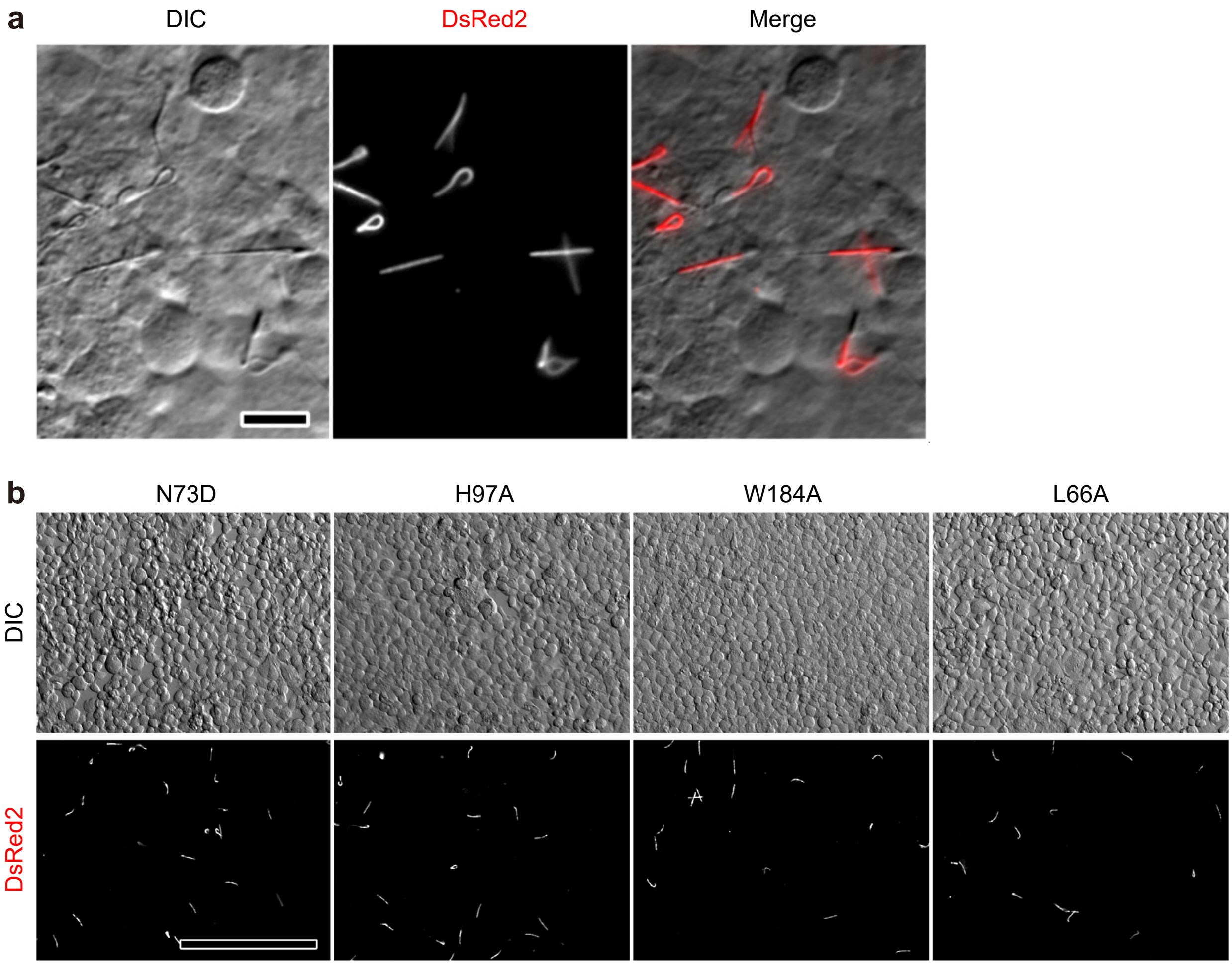

Supplementary Figure 5 | Sperm counting on JUNO-expressing cultured cells.
(**a**) Binding of spermatozoa with mitochondrial DsRed2 to mJUNO-expressing HEK293T cells. Light, fluorescence microscopic and merged images of spermatozoa bound to mJUNO-expressing HEK293T cells are shown. Bar indicates 20 μm.
(**b**) Binding of spermatozoa with mitochondrial DsRed2 to HEK293T cells expressing mutant mJUNOs. Light (upper image) and fluorescence (lower image) microscopic images of transfected cells after insemination with fluorescent spermatozoa are shown. Each picture corresponds a quarter area of the field used for analysis. Bar indicates 200 μm.
